# A Longitudinal Study on the Self-Reported Confidence of Medicine Interns Regarding Interpreting Chest Radiographs and Electrocardiograms, Performing Procedures, and Communicating With Patients' Families

**DOI:** 10.7759/cureus.105246

**Published:** 2026-03-14

**Authors:** Saurabh Dubey, Matthew Roland, Karen Beekman

**Affiliations:** 1 Internal Medicine, Flushing Hospital Medical Center, New York City, USA; 2 Internal Medicine, State University of New York Downstate Medical Center, New York City, USA; 3 Biostatistics, Flushing Hospital Medical Center, New York City, USA

**Keywords:** communicating with families, community teaching hospital, confidence, higher education medical training, longitudinal education, medical intern, reading chest radiographs, reading ekgs

## Abstract

Background

Gaining confidence in the performance of basic medical procedures and patient communication is integral to resident training. Research suggests that residents tend to exhibit more confidence over the course of their training, but findings have been mixed regarding different competency domains. Furthermore, changes in confidence throughout the intern year of residency remain underexplored.

Objective

This study aimed to assess changes during the intern year in self-reported confidence in chest radiograph interpretation, electrocardiogram (EKG) interpretation, basic procedures, and communication with families and to identify domains needing targeted training. All four domains were considered co-primary for this study.

Methods

This exploratory survey study assessed the confidence of 21 of 27 (78%) medical residents training at a community hospital on the East Coast of the United States. Surveys were provided at three timepoints across 10 months from 2024 to 2025 (at the start and at the four- and 10-month follow-ups).

Results

Eight residents completed at least two of the surveys (38%). Significant differences in confidence across the timepoints were seen for chest X-ray interpretation (H=6.43; p=0.04) and performing basic procedures (H=7.61; p=0.02). No significant differences were seen for EKG interpretation or communication with families (all p>0.05). Repeated measures assessments were inconclusive (all p>0.05).

Conclusion

These findings highlight a need for training targeting EKG interpretation and performing basic procedures. Providing more hands-on, interactive training for interns may facilitate confidence and preparedness earlier in the training process.

## Introduction

Medical residency programs aim to shape students into competent, independent practitioners [1]. The official Accreditation Council of Graduate Medical Education (ACGME) identifies six core competencies that are essential for medical intern training: Patient Care, Medical Knowledge, Practice-Based Learning and Improvement, Interpersonal and Communication Skills, Professionalism, and Systems-Based Practice [2]. Communicating effectively with patients and their families falls into three of these core competencies: Patient Care, Interpersonal and Communication Skills, and Professionalism. Performing procedures falls into the domain of Practice-Based Learning and Improvement. Furthermore, according to the ACGME Internal Medicine Milestones framework, trainees are expected to understand the indications for and demonstrate basic interpretation of common diagnostic testing, explicitly including electrocardiograms (EKGs) and chest radiographs as part of the Medical Knowledge subcompetency for diagnostic testing [3].

Medical interns' confidence to independently perform procedures, communicate with patients and families, and interpret clinical findings from radiographs or EKGs was evaluated in this study. These parameters were chosen for this study because some are part of the ACGME core competency requirements, they are skills applicable to a wide range of specialties (not all medical interns go on to specialize in Internal Medicine with some of them going on to specialize in other fields like Anesthesiology where such skills are also applicable), or they represent fundamental methods for diagnosing potentially life-threatening conditions as outlined below. 

Chest radiograph and EKG interpretation are essential skills for diagnosing potentially life-threatening conditions such as pneumothorax, pneumonia, or ST-segment elevation myocardial infarction (STEMI) [4-13]. Despite the importance of certainty and confidence in interpretations, research shows that residents' confidence can be mixed throughout their training. Some studies have reported increased confidence throughout training [14], whereas others have shown low confidence and accuracy by the final years and post-residency [15,16].

Similarly, procedural skills such as venipuncture, central line placement, and other bedside techniques are expected to develop early in training [17]. However, research has shown that many enter residency with minimal exposure to such procedures, resulting in uncertainty and discomfort performing them [18].

Effective communication with patients and families is another crucial skill that greatly affects patient satisfaction and outcomes [19]. Confidence in communication generally improves with experience, but many residents report less confidence early in their training, and improvement can be slow without structured training or guidance [20-23].

Despite existing research tracking confidence over the course of residency, few studies have explored changes during the intern year. Furthermore, resident confidence in community hospital settings, where educational resources may be limited, has not been explored. This exploratory study addresses these gaps by following Internal Medicine residents at a community hospital on the East Coast of the United States and assessing self-perceived confidence throughout their intern year. We aim to identify areas where targeted interventions can be implemented to enhance the training and preparedness of Internal Medicine interns in these basic areas of core competencies. 

Our prediction before the study was conducted was that self-reported confidence would increase when measured over time in all four domains as we expected confidence to increase with more hands-on training and practice. 

## Materials and methods

This exploratory survey study followed a group of 27 Internal Medicine residents from Flushing Hospital Medical Center, a community hospital in Flushing, New York, during their intern year from July 2024 to April 2025. Surveys were administered electronically through a Health Insurance Portability and Accountability Act (HIPAA)-compliant SurveyMonkey account to residents at the start of the cohort, at the four-month follow-up, and at the 10-month follow-up. The data was collected at the start as a baseline. The timepoints of four months and 10 months were chosen to form a basis for intervals of possible targeted intervention.

Informed consent was requested at the start of each administration. To link participant responses across timepoints, respondents were asked to list their first and last initials on each survey. Surveys included four items assessing confidence speaking with families, interpreting EKGs and chest radiographs, and performing basic medical procedures (e.g., phlebotomy, central line placement, and arterial puncture). These items were measured on a 3-point Likert scale ranging from "Not at all" to "Very confident". Other items assessed whether residents had experience performing procedures prior to their rotations and their willingness to see patients before morning reports.

Data were analyzed using the R software (R Foundation for Statistical Computing, Vienna, Austria). Due to inconsistent follow-up and new respondents at later timepoints, the sample was analyzed as a repeated cross-sectional sample. Confidence scores were treated as an ordinal variable and analyzed using non-parametric methods due to the ordinal Likert scale structure of the data and small sample size [24]. Participants who did not respond to any confidence items were excluded from confidence item analyses. Imputation was not used to account for missing data, and all analyses performed were conducted with available cases. Confidence scores were analyzed on a continuous scale from 1 to 3. Analyses used non-parametric methods due to the ordinal Likert scale structure of the data and small sample size. Due to low follow-up rates and the repeated cross-sectional design of this study, Kruskal-Wallis tests with Dunn post-hoc comparisons using Holm adjustments performed for significant omnibus tests were used to assess between-group differences in confidence among the three timepoints [25]. Secondary, exploratory longitudinal investigation of within-person changes in confidence among the eight participants who had at least one follow-up was measured using Skillings-Mack tests to accommodate missing follow-up observations across timepoints [26,27]. Test statistics, including H or χ² for Kruskal-Wallis or Skillings-Mack, respectively, degrees of freedom, medians and interquartile ranges (IQR), and p-values were reported. Epsilon-squared (ε^2^ _R_) effect sizes were reported for Kruskal-Wallis tests. Two-sided p-values of <0.05 were considered statistically significant.

This study was reviewed and approved by the Flushing Hospital Medical Center Institutional Review Board (approval number: 2202824-1) on July 9, 2024. The research was conducted in accordance with the ethical principles outlined in the Declaration of Helsinki (1975, as revised in 2005) and in compliance with applicable federal regulations governing human subjects research (45 CFR Part 46). Written informed consent was obtained from all participants prior to enrollment.

Participant confidentiality was strictly maintained throughout the study. No directly identifying information was collected as part of the survey. Survey responses were recorded using a secure, password-protected electronic platform with access restricted to authorized study investigators only. Data were stored on encrypted, institutionally secured servers, and all study files were de-identified prior to analysis. Only aggregate data are reported in this manuscript. A sample copy of the consent form and survey instrument are provided in Appendix A and Appendix B.

## Results

Across the three survey administration periods, 27 respondents were recorded. 55.6% responded to the baseline survey (n=15), 55.6% responded on the four-month follow-up (n=15), and 40.7% responded to the 10-month follow-up (n=11). Of the 15 respondents on the baseline survey, 33.3% (n=5) reported having experience performing basic medical procedures prior to beginning internship. Descriptive analyses indicated a trend toward fewer residents seeing patients before morning reports by timepoint 3. Specifically, 12.5% reported not having time to see patients prior to their morning reports at baseline, but this proportion increased to 33% by the 10-month follow-up.

Missingness broadly affected confidence items. Participants who did not answer any questions pertaining to confidence were excluded from further analysis. Four respondents at baseline, three at four months, and one at 10 months omitted all confidence items and were excluded from further analyses for those timepoints. The final analytic sample included 12 participants at baseline, 12 at the four-month follow-up, and 11 at the 10-month follow-up. All participants who were assessed answered all confidence items, so no item-level missingness impacted analyses within timepoints. Only eight participants completed at least one follow-up survey.

**Figure 1:**
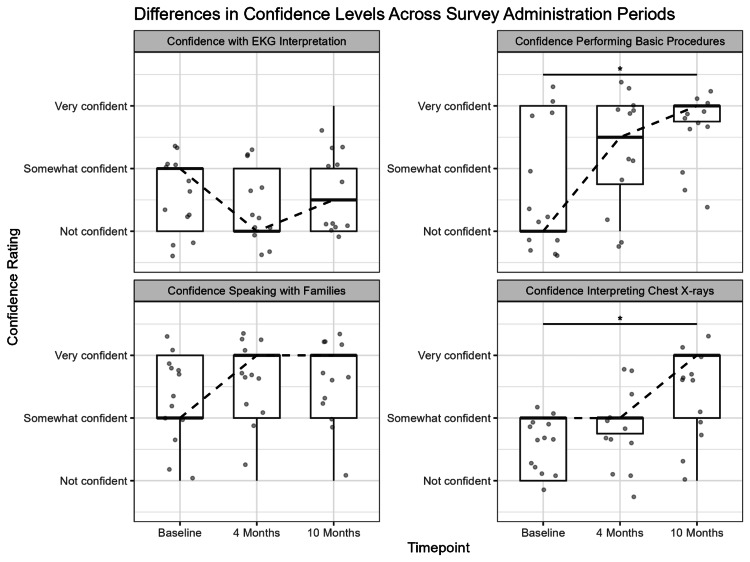
Representation of median differences in confidence levels across the three timepoints Asterisks represent significant differences observed through Kruskal-Wallis testing. EKG: electrocardiogram

Median confidence scores are displayed in Table 1, with distributions summarized as boxplots in Figure 1. Results indicated significant differences among timepoints for confidence with radiograph interpretation (H=6.43; p=0.04; df=2; ε^2^ _R_=0.19) and performing basic procedures (H=7.61; p=0.02; df=2; ε^2 ^_R_=0.22). The Dunn post-hoc testing revealed that, for both domains, confidence was significantly higher at the 10-month follow-up compared to baseline (basic procedures: p=0.02; radiograph interpretation: p=0.03). However, confidence levels were not significantly different between baseline and the four-month follow-up (basic procedures: p=0.08; radiograph interpretation: p=0.26) or from the four months to 10 months (basic procedures: p=0.30; radiograph interpretation: p=0.15).

**Table 1:** Median confidence levels by timepoint Values are presented as median (IQR). Higher scores indicate greater self-reported confidence. Kruskal-Wallis tests evaluated between-group differences. Skillings-Mack tests evaluated within-person changes among residents with repeated observations. P-values of <0.05 are considered statistically significant. *p<0.05

Domain	Timepoint 1 (N=12)	Timepoint 2 (N=12)	Timepoint 3 (N=11)	Overall (N=35)	H(df), p	χ²(df), p
Speaking with families	2.00 (1.00-3.00)	3.00 (1.00-3.00)	3.00 (1.00-3.00)	3.00 (1.00-3.00)	H(2)=1.49; p=0.48	χ²(2)=2.08; p=0.35
Electrocardiogram interpretation	1.50 (1.00-2.00)	1.00 (1.00-2.00)	1.00 (1.00-2.00)	1.00 (1.00-2.00)	H(2)=0.16; p=0.92	χ²(2)=0.66; p=0.72
Basic medical procedures	1.00 (1.00-2.25)	2.50 (1.75-3.00)	3.00 (2.50-3.00)	2.00 (1.00-3.00)	H(2)=7.61; p=0.02*	χ²(2)=3.04; p=0.22
Radiograph interpretation	2.00 (1.00-2.00)	2.00 (1.75-2.00)	3.00 (2.00-3.00)	2.00 (1.00-2.00)	H(2)=6.43; p=0.04*	χ²(2)=3.04; p=0.07

No significant differences in confidence levels for speaking with families (H=1.49; p=0.48; df=2; ε^2^ _R_=0.04) or EKG interpretation (H=0.16; p=0.92; df=2; ε^2^ _R_=0.005) were observed. As shown in Table 1 and Figure 1, the median confidence speaking with families was consistently high across all timepoints, whereas the median confidence interpreting EKGs was consistently low across timepoints.

Among the eight respondents with longitudinal data, Skillings-Mack tests found no significant differences in confidence interpreting chest X-rays (χ2=3.04; p=0.07), performing basic procedures (χ2=3.04; p=0.22), communicating with families (χ2=2.08; p=0.35), or interpreting EKG findings (χ2=0.66; p=0.72).

## Discussion

Results of this exploratory study indicate that intern confidence tends to be higher near the end of training for performing basic procedures and X-ray interpretation, with moderate to large effect sizes, suggesting incremental improvement in confidence by the end of the intern period. No differences among timepoints were observed for EKG interpretation or communication with families. Within-person changes in intern confidence were not significant. This could be a result of low power due to inconsistent follow-ups.

Confidence for EKG interpretation was low across all timepoints, and no residents rated themselves as "very confident" in this domain. This could reflect the complexity of EKG findings or the need for improved training. Surprisingly, confidence for communicating with families was consistently high across all timepoints, with many residents reporting feeling "very confident" with their communication skills. This finding contrasts with the reported learning curves associated with effective communication with patients' families. However, this may reflect limitations of the scale used to assess this domain. Finally, confidence in performing basic procedures did increase over time, but as shown in Table 1 and Figure 1, confidence tended to be variable with a large interquartile range at baseline.

Findings suggest a need for enhanced EKG interpretation training and more structured support for performing basic medical procedures during internship. Possible solutions include dedicated EKG interpretation sessions, simulation-based training, or increased faculty mentorship during EKG review. Previous research has demonstrated improved performance following the implementation of interactive training programs. A study conducted on French medical students showed that a "reversed classroom" method (in which students presented EKGs and their interpretations to each other under a cardiologist's supervision) was found to be more effective than a conventional lecture-based teaching model [28]. 

Limitations

Our sample size was small because of using a single-center, intern cohort, limiting statistical power.

Selection bias may have occurred across timepoints. Participation was voluntary, and interns who chose to respond across timepoints may have differed from those who later became non-respondents. Furthermore, low response rates, particularly at the end of the study period, could lead to possible non-respondent bias. If more motivated or academically confident interns were more likely to complete surveys, overall confidence levels may be overestimated.

Confidence was measured using a 3-point Likert scale, which may have limited sensitivity and introduced ceiling or floor effects. Future studies could use a more granular 5- or 7-point Likert scale to better capture variation in resident confidence. Due to these limitations, our primary analyses were performed at the between-group level, which could result in model misspecification, as eight participants did have follow-up data. Furthermore, analyses were unadjusted because of the limited sample size. Thus, our findings should be interpreted cautiously. Additionally, data collection concluded 10 months into the intern year, possibly neglecting further improvement that could occur in the last two months. Given the exploratory nature of the study, no formal multiplicity correction was applied across domains; thus, p-values should be interpreted cautiously.

The study was also conducted only at a single site, a community hospital in a large city, and so the findings may not be as applicable to other settings, such as tertiary care centers or rural community hospitals. This is because of both differing learning environments and variability in the pool of candidates who apply to hospitals in different settings. 

Finally, the study evaluated self-reported confidence, which can be subjective compared to more objective metrics of capabilities. It is very possible to be confident about interpreting a chest radiograph or EKG and for that interpretation to be wrong. Studies on the correlation between self-reported confidence and clinical competence show only a weak correlation. Brinkman et al. found only a weak positive correlation between self-reported prescribing skills in a group of 403 fourth-year medical students [29]. Another study on final-year medical students actually showed a negative correlation between self-reported confidence and objective competence when it came to performing procedures [30]. 

All of these limitations should be addressed in future work evaluating changes in intern confidence. Future research can address these limitations by incorporating larger sample sizes using multi-center cohorts. Furthermore, studies can incorporate more objective assessments of physician competency and confidence. Future research can address potential confounders, such as prior experience and exposure, for expanded, adjusted modelling.

## Conclusions

Our findings suggest that interns may experience improvements in self-reported confidence in interpreting chest radiographs and performing certain basic procedures over differing timepoints in their training period. However, confidence in interpreting EKGs did not appear to improve, and confidence in performing basic procedures varied, indicating potential areas for curricular enhancement. Given the exploratory design and reliance on self-reported measures, these results should be interpreted as preliminary. Further studies with larger samples and objective assessments are needed to better evaluate changes in competency and training effectiveness.

## Figures and Tables

**Figure 2:**
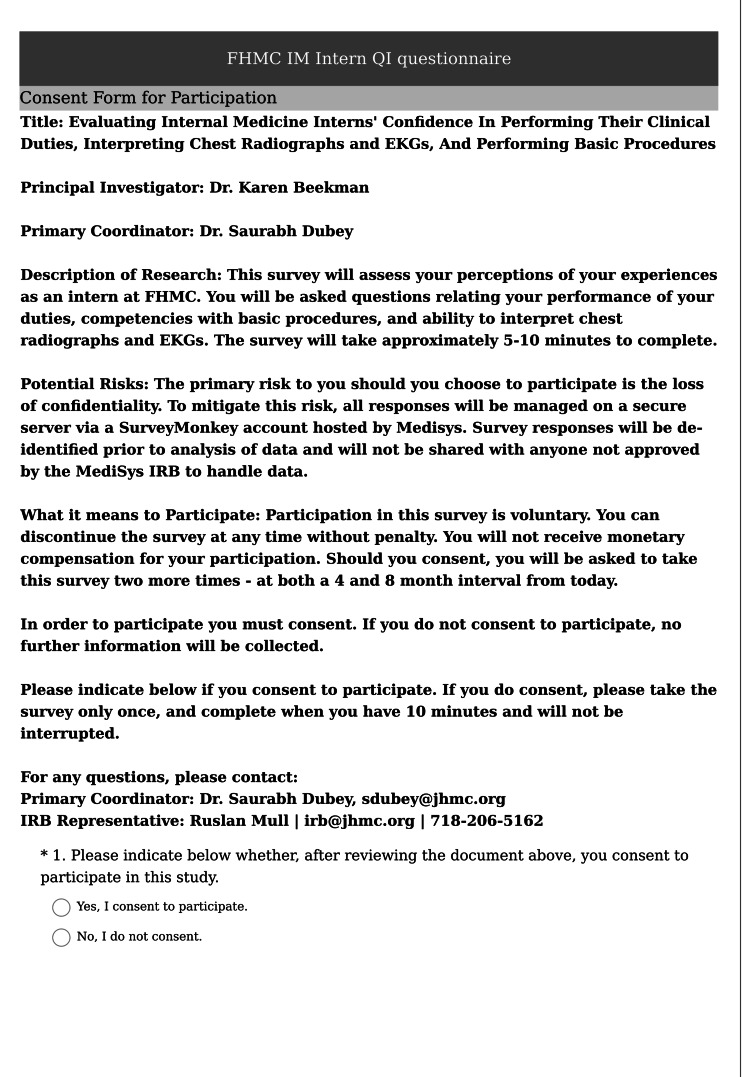
Sample of the consent form used in the study (page 1/2)

**Figure 3:**
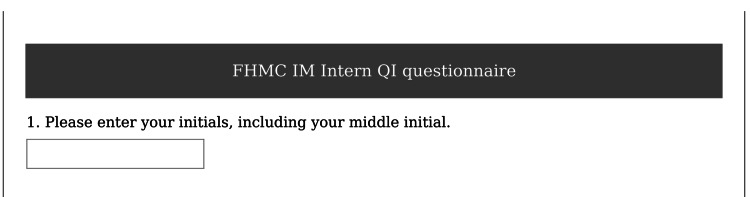
Sample of the consent form used in the study (page 2/2)

**Figure 4:**
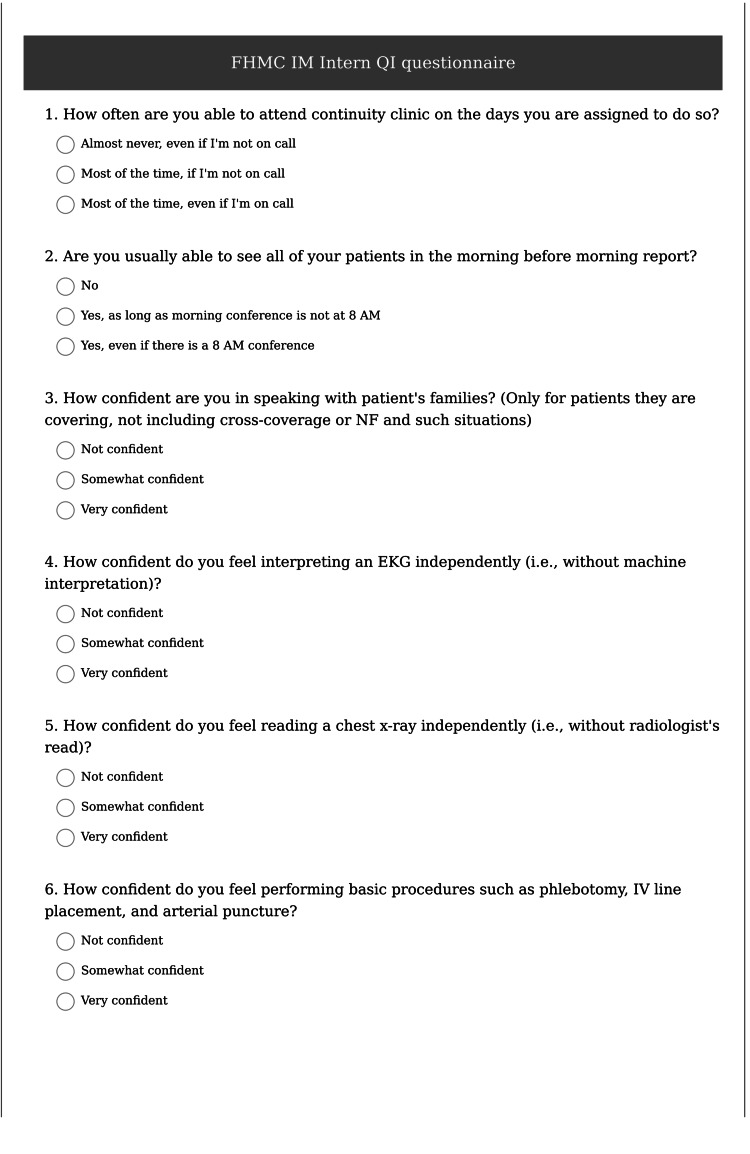
Sample questionnaire used in the study

## Appendices

Appendix A

Appendix B
